# Alteration in maternal serum uric acid levels in pre-eclampsia and associated perinatal outcomes: a cross-sectional study in Ghana

**DOI:** 10.11604/pamj.2024.47.49.37106

**Published:** 2024-02-07

**Authors:** Kwame Adu-Bonsaffoh, Daniel Quarshie Kudaya, Bayor Fidelis, Linda Ahenkorah Fondjo, John Ahenkorah

**Affiliations:** 1 Department of Obstetrics and Gynaecology, University of Ghana Medical School, Accra, Ghana; 2 Department of Obstetrics and Gynaecology, Korle Bu Teaching Hospital, Accra, Ghana; 3 Department of Physiology, University of Ghana Medical School, Accra, Ghana; 4 Department of Molecular Medicine, School of Medical Sciences, Kwame Nkrumah University of Science and Technology, Kumasi, Ghana; 5 Department of Anatomy, University of Ghana Medical School, Accra, Ghana

**Keywords:** Uric acid, hyperuricemia, pre-eclampsia, Ghana

## Abstract

**Introduction:**

pre-eclampsia (PE) is a multisystemic pregnancy-specific hypertensive disorder associated with significant adverse maternal and perinatal outcomes. Maternal serum uric acid level is hypothesized as a reliable marker for predicting the severity and adverse outcomes of pre-eclampsia and facilitating clinical decisions. This study explored the association between maternal serum uric acid and adverse pregnancy outcomes in pre-eclampsia.

**Methods:**

a cross-sectional study involving women diagnosed with pre-eclampsia was conducted at Korle-Bu Teaching Hospital (KBTH), a tertiary hospital in Ghana. Descriptive analyses were performed and multivariable logistic regression model was used to explore the association between maternal serum uric acid levels and pregnancy outcomes using R software.

**Results:**

we included 100 women with pre-eclampsia comprising 79% and 21% preterm and term pre-eclampsia respectively and with mean gestational age (GA) at diagnosis of 32.35±2.66 weeks and 35.96±1.94 weeks respectively. The mean maternal age of preterm and term pre-eclampsia groups was 29.81±5.29 years and 29.46±5.78 years respectively. Hyperuricemia (serum uric acid >375 µmol/L) occurred in 61% of the pre-eclamptic women. The mean gestational age (in weeks) at diagnosis was significantly lower in the pre-eclamptic women with hyperuricemia compared with those with normal levels of uric acid (33.51±3.03 versus 34.80±2.71). There was a significant negative association (moderate correlation) between maternal serum uric acid levels and birth weight (R= -0.34, p < 0.001) in pre-eclampsia; the statistical significance was limited to preterm only (Pearson R= -0.39, p-value <0.001) but not term pre-eclampsia. Hyperuricemia was significantly associated with low birth weight [aOR: 3.222 (95% CI: 1.098, 10.393)], caesarean section [aOR: 2.281 (95% CI: 1.084, 7.568)] and severe diastolic pressure at birth [aOR: 3.517 (95% CI: 1.123, 11.939)].

**Conclusion:**

hyperuricemia in pre-eclampsia was significantly associated with both maternal (caesarean section and severe hypertension) and neonatal (low birth weight) adverse outcomes. Hyperuricemia seems clinically useful in predicting pregnancy outcomes, especially in preterm pre-eclampsia. Further longitudinal study is recommended in exploring the clinical significance of maternal uric acid levels and pregnancy outcomes in pre-eclampsia.

## Introduction

Pre-eclampsia (PE) is a multisystemic pregnancy-specific disorder, comprising new onset of hypertension and proteinuria in a previously normotensive woman after the 20^th^ week of gestation [1]. It is the most severe and prevalent form of hypertensive disorder in pregnancy (HDP) associated with adverse perinatal outcomes. Globally, pre-eclampsia accounts for between 2 to 8% of all pregnancies with remarkably high maternal and neonatal complications [2]. In Ghana, HDP including pre-eclampsia remains the leading cause of maternal mortality in tertiary hospitals and accounts for about 18% of maternal deaths in the country [3].

The main pathophysiologic mechanism of PE remains unknown despite extensive research globally [4]. Inadequate knowledge of the etiology of PE has resulted in a lack of clinical consensus on the optimal therapeutic interventions to improve pregnancy outcomes. To date, delivery of the baby and placenta remains the most consistent clinical intervention to reduce maternal and fetal complications [5]. The decision to deliver the fetus is clinically challenging especially in women with extremely preterm gestation due to increased risks of prematurity and perinatal death. However, several biomarkers and clinical indicators have been devised and applied clinically to provide enough evidence of the disease's severity. Traditionally, maternal serum uric acid levels in addition to other pre-eclampsia tests have been employed to predict pre-eclampsia severity and facilitate clinical decision [6]. Uric acid is a heterocyclic compound produced from purine metabolism and synthesized by the enzyme xanthine oxidase. The production of uric acid by xanthine oxidase is coupled with the production of free radical superoxide which has a causal association with oxidative stress [7].

In normotensive pregnancies, the glomerular filtration rate increases resulting in enhanced renal clearance and a reduction in serum uric acid levels by up to 35% due to increased secondary and or decreased proximal tubular absorption and a decline in its production [8]. On the other hand, hyperuricemia is a common feature of severe pre-eclampsia and clinically precedes the development of hypertension and proteinuria. Hyperuricemia is traditionally considered a biomarker of renal damage primarily from reduced renal tubular excretion [7]. In pre-eclampsia, uric acid serves as a marker of disease severity and a reliable predictor of pregnancy outcomes [6,9]. Uric acid contributes to failed placenta bed remodeling by impeding trophoblast invasion and reducing placental perfusion resulting in ischemic reperfusion injury to the placenta and oxidative stress. With tissue injury, purines are released and with hypoxia, ATP is converted to adenine and xanthine (substrate). Further hypoxia leads to the stimulation of xanthine oxidase and increased production of uric acid. Furthermore, vasospasm and increased loss of fluid due to endothelial dysfunction also stimulate renal absorption of uric acid and further compound the production of uric acid and a reduction in its excretion [7].

The association between serum uric acid and complications of pre-eclampsia is still conflicting [8,9]. In a systematic review, Thangaratinam *et al*. determined a significant association between hyperuricemia and adverse pregnancy outcomes including severe pre-eclampsia, eclampsia, and perinatal death [10]. More recently, Priya *et al*. demonstrated that hyperuricemia is positively associated with fetal but not maternal complications [11]. Also, Bellos *et al*. recently reported up to 82% sensitivity of hyperuricemia in predicting adverse perinatal outcomes and specificity of about 70% [12]. In Ghana, the clinical significance of uric acid in determining the severity and pregnancy outcomes remains unclear. In a previous study, we reported a significant reduction and increment in maternal uric acid levels in normotensive pregnancies and pre-eclampsia respectively compared with non-pregnant women [13]. This study further explores the association between maternal serum uric acid and adverse perinatal outcomes in pre-eclampsia.

## Methods

**Study design and settings:** this is a secondary analysis of a cross-sectional study conducted at the Maternity unit of the Korle Bu Teaching Hospital (KBTH) in Accra, Ghana between April and June 2011. Korle Bu Teaching Hospital is the largest teaching hospital in Ghana with approximately 10,000 deliveries per year. The detailed methodology has been described elsewhere [13,14].

**Participants:** the inclusion criteria consisted of pregnant women between 18 to 42 years old with a confirmed diagnosis of pre-eclampsia. All the pre-eclamptic women included in the study had performed an ultrasound scan in the first half of their gestations for accurate pregnancy dating. All the included women had single gestations. Specific exclusion criteria were women with multiple gestations and chronic medical conditions such as chronic hypertension, diabetes, sickle cell disease, renal disease, and cardiac disease in pregnancy.

**Variables:** the primary outcome was hyperuricemia in pre-eclampsia, defined as maternal serum uric acid levels ≥375 µmol/L (≥6.3mg/dl) [15]. The secondary outcomes were low birth weight (birth weight <2500g), severe blood pressure (BP) at birth (systolic BP≥160mmHg, diastolic BP≥110mmHg), and mode of delivery (vaginal route or caesarean section). Pre-eclampsia was categorized into preterm and term phenotypes. Pre-eclampsia was defined as new onset of systolic blood pressure ≥140 mm Hg and diastolic blood pressure ≥90 mm Hg after 20 weeks of gestation, with associated proteinuria of ≥300mg in the 24-hour urine sample or a random dipstick of +1 or more [16]. Preterm pre-eclampsia was defined as pre-eclampsia diagnosed before 37 weeks of pregnancy and term pre-eclampsia as occurring at or after 37 weeks [17]. Severe pre-eclampsia was defined as pre-eclampsia as a systolic blood pressure greater than or equal to 160 mm Hg and/or a diastolic blood pressure greater than or equal to 110 mmHg [18]. Adverse maternal morbidities (caesarean section as a mode of delivery, severe systolic and diastolic blood pressure at the time of birth) and perinatal outcomes (birth weight, Apgar scores at 1 and 5 minutes) outcomes were assessed. Other maternal variables assessed include maternal age in years, parity (number of previous births), gestational age at booking and delivery, and blood pressure (mmHg) at booking and birth.

**Data sources/measurement:** a detailed description of the study procedure has been published [13,14]. Briefly, following the recruitment of the study participants and informed consent, they were interviewed using structured questionnaires to document their demographic and obstetric characteristics. From their medical records, we also extracted the relevant medical information such as blood pressure maternal age, and parity. After birth, neonatal data were also obtained from the medical records of the participants and these included the Apgar scores, birth weights in grams, gestational age at birth in weeks, and blood pressure in mmHg at the time of birth.

**Blood sampling:** venous blood samples of about 5mls were taken from the cubital fossa after the application of a tourniquet under aseptic conditions. The blood samples were taken at the time of presentation before the initiation of antihypertensive treatments or magnesium sulfate protocol. The blood was collected into sterile sample tubes devoid of coagulation inhibitors. The samples were then centrifuged at approximately 1000xg for 15 mins at 4 degrees Celsius. The serum was decanted and stored at a temperature of -80 C until the laboratory assays were done. The sera were then analyzed using biosystems reagents, in a URA semi-automated chemistry analyzer (Medsource Ozone Biomedicals) for parameters pertinent to pre-eclampsia such as uric acid, urea and creatinine.

**Sample size and bias:** in this secondary analysis, we included all the women with confirmed diagnoses of pre-eclampsia from the primary study. The sampling methods have previously been described [13,14]. To ensure accurate diagnosis and reduce selection bias, all the pregnant women with pre-eclampsia had ultrasound scans performed before 20 weeks of gestation. Also, serum measurement of uric acid was undertaken strictly based on standardized protocols to ensure accurate results.

**Statistical analysis:** the data were entered into a Microsoft Excel spreadsheet (version 2003). The data analyses were done using R software (version 3.6.3, R Core Team, Vienna, Austria). Descriptive analyses were initially conducted and results were presented in mean± standard deviation (SD) and percentages. The pre-eclampsia population was categorized into preterm (< 37 weeks) and term (≥37 weeks) groups based on the timing of the initial diagnosis. Student t-test was used to assess the association between preterm and term pre-eclampsia for normally distributed continuous variables. The Chi-square test (or Fisher exact test where appropriate) was performed to assess the association between the categorical variables. Maternal serum uric acid levels were categorized into normal (<375 µmol/L), and high or hyperuricemia (≥375 µmol/L) [15]. A multivariable logistic regression model was used to explore the association between maternal serum uric acid levels (hyperuricemia) and birth weight, mode of birth, and blood pressure at diagnosis. We considered p value <0.05 as significant.

**Ethical consideration:** the ethical approval was obtained from the Ethics and Protocol Review Committee of the University of Ghana Medical School (Protocol Identification Number: MS-Et/M.4-P.5.5/2010-11). Informed consent was taken from all participants after fulfilling the inclusion criteria. The study participants were informed that participation in the study was purely voluntary and failure to participate did not affect the quality of healthcare they would receive, in addition, the participants were told they could also withdraw from the study at any time.

**Funding:** this study received funding from the College of Health Sciences, University of Ghana in the form of a research grant for postgraduate studies for Dr Kwame Adu-Bonsaffoh. The funders had no contribution to the study design, data collection/analysis, preparation of the manuscript, or the decision to publish.

## Results

**Figure 1:**
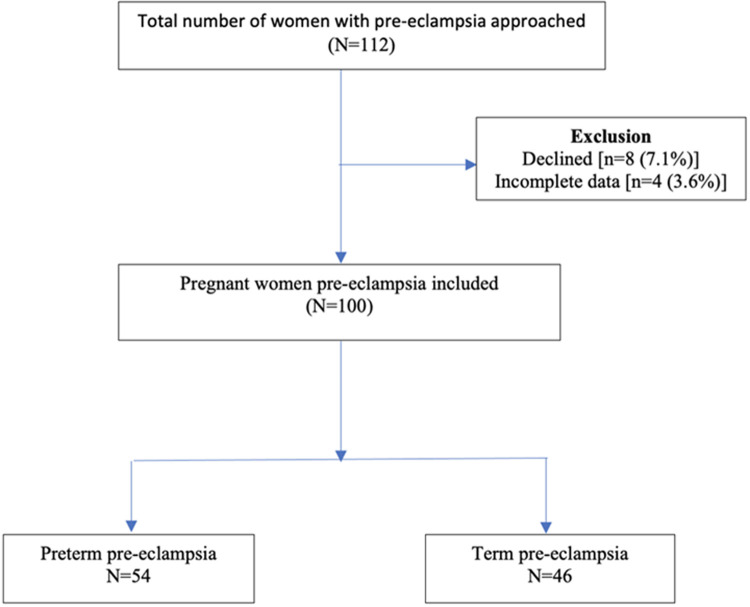
flow chart indicating the recruitment of study participants

In this study, 112 women with pre-eclampsia were initially approached and 8 (7.1%) declined to participate. In addition, we exclude 4 (3.6%) women on account of incomplete data. We therefore included 100 (89.3%) pregnant women with pre-eclampsia in the study comprising 79 and 21 preterm and term pre-eclampsia respectively (Figure 1). The mean maternal ages of preterm and term pre-eclampsia groups were 29.81 ± 5.29 years and 29.46 ± 5.78 years respectively. The mean gestational age (GA) at diagnosis of the preterm PE group was 32.35 ± 2.66 weeks compared to the term PE group of 35.96 ± 1.94 weeks. No significant difference was detected concerning parity, serum urea, creatinine, and uric acid levels between preterm and term pre-eclampsia groups. Both systolic and diastolic blood pressures at birth were significantly higher in preterm compared to term pre-eclampsia (Table 1). However, the two groups had no significant differences concerning the systolic and diastolic blood pressures at booking and diagnosis.

**Table 1:** general characteristics and pregnancy outcomes of women with preterm and term pre-eclampsia

Characteristic	Preterm pre-eclampsia group (n=54); mean±SD	Term pre-eclampsia group (n=46); mean±SD	P-value
Maternal age (years)	29.81±5.29	29.46±5.78	0.172
Parity	0.81±1.15	0.67±1.27	0.105
GA at booking (weeks)	19.17±5.20	19.14±6.24	0.988
GA at diagnosis (weeks)	32.35±2.66	35.96±1.94	0.000
sBP at booking (mmHg)	109.97±9.18	111.33±7.83	0.501
dBP at booking (mmHg)	67.91±7.49	70.29±5.98	0.134
SBP at diagnosis (mmHg)	169.78±24.72	160.85±14.27	0.087
DBP at diagnosis (mmHg)	109.41±12.68	102.61±9.29	0.139
Serum urea (mg/dl)	6.24±2.87	6.91±2.93	0.801
Serum creatinine (µmol/L)	113.24±31.74	117.23±50.83	0.497
Serum uric acid (µmol/L)	385.87±94.97	354.54±89.08	0.167
**Pregnancy outcomes**			
GA at birth (weeks)	34.56±1.83	37.52±0.62	0.000
sBP at birth (mmHg)	180.43±17.09	171.91±12.68	0.026
dBP at birth (mmHg)	113.01±10.03	107.14±7.84	0.007
Birth weight	2217.72±646.39	3190.95±357.52	<0.001
APGAR 1	6.15±1.68	6.71±1.77	0.202
APGAR 5	7.27±1.80	7.71±2.00	0.364
Mode of delivery^m,n^			0.037
Vaginal birth	17 (21.8)	10 (47.6)	
Caesarean section	61 (78.2)	11 (52.4)	
Low birth weight^n^			<0.001
No	30 (38.0)	21 (100)	
Yes	49 (62.0)	0	
sBP at birth^n^			0.322
Mild	4 (5.1)	3 (14.3)	
Severe	75 (94.9)	18 (85.7)	
dBP at birth^n^			0.028
Mild	11 (13.9)	8 (38.1)	
Severe	68 (86.1)	13 (61.9)	

m missing=1; ^n^results presented in number (%); GA=gestational age; sBP=systolic blood pressure; dBP=diastolic blood pressure

Among the preeclamptic women, 72% delivered via caesarean section and this was significantly higher in the preterm compared with the term pre-eclampsia groups 78.2% versus 52.4%). Women with preterm pre-eclampsia had significantly lower mean birth weight compared with the term pre-eclampsia (2217.72 ± 646.39g versus 3190.95 ± 357.52). Low birth weight occurred in 49% of the neonates. There were no significant differences between preterm and term pre-eclampsia concerning APGAR cores at one and five minutes (Table 1).

**Hyperuricemia and pre-eclampsia:** hyperuricemia (serum uric acid >375 μmol/L) occurred in 61% of the pre-eclamptic population with no significant difference between preterm and term categories [50 (63.3%) versus 11 (52.4%)]. The mean gestational age at diagnosis was significantly lower in the pre-eclamptic women with hyperuricemia compared with those with normal levels of uric acid (33.51 ± 3.03 versus 34.80 ± 2.71). Also, pre-eclamptic women with hyperuricemia had lower gestational age at delivery compared to the normo-uricemia group although this did not reach statistical significance (36.38 ± 1.94 versus 35.62 ± 2.07). Pre-eclampsia hyperuricemia was associated with a statistically significant lower birth weight than normo-uricemia (2651 ± 609.17 g versus 2275.41 ± 746.02 g). Table 2 shows the comparison of normal uric acid levels and hyperuricemia in pre-eclampsia in terms of maternal features and pregnancy outcomes.

**Table 2:** maternal characteristics and pregnancy outcomes of hyperuricemia in pre-eclampsia

Characteristic	Normal uric acid levels (n=39); mean±SD	Hyper-uricemia(n=61); mean±SD	P-value
Maternal age (years)	29.41±5.93	29.79±5.22	0.747
Parity	0.74±1.14	0.75±1.25	0.966
GA at booking(weeks)	19.31±6.01	19.07±5.03	0.835
GA at diagnosis (weeks)	34.80±2.71	33.51±3.03	0.029
sBP at booking (mmHg)	110.87±8.06	109.87±9.44	0.572
dBP at booking (mmHg)	68.71±6.95	68.21±7.46	0.732
sBP at diagnosis (mmHg)	166.62±22.32	165.07±20.22	0.727
dBP at diagnosis (mmHg)	107.38±13.93	105.571±10.09	0.485
Serum urea (mg/dl)	6.95±3.10	6.29±2.76	0.280
**Pregnancy outcomes**			
GA at birth (weeks)	36.38±1.94	35.62±2.07	0.066
sBP at birth (mmHg)	176.41±17.84	180.07±16.17	0.304
dBP at birth (mmHg)	129.51±8.73	122.00±10.40	0.087
Birth weight	2651±609.17	2275.41±746.02	0.007
APGAR 1	6.50±1.74	6.13±1.69	0.302
APGAR 5	7.56±1.96	7.25±1.78	0.434
Mode of delivery^m^			0.025
Vaginal birth^n^	16 (41.0)	11 (18.3)	
Caesarean section^n^	23(59.0)	49 (81.7)	
Low birth weight			0.059
No	25 (64.1)	26 (42.6)	
Yes	14 (35.9)	35 (57.4	
sBP at birth			0.556
Mild	4 (10.3)	3 (4.9)	
Severe	35 (89.7)	58 (95.1)	
dBP at birth			0.033
Mild	12 (30.8)	7 (11.5)	
Severe	27 (69.2)	54 (88.5	

m missing=1; ^n^results presented in number (%); GA=gestational age; sBP=systolic blood pressure; dBP=diastolic blood pressure

**Figure 2:**
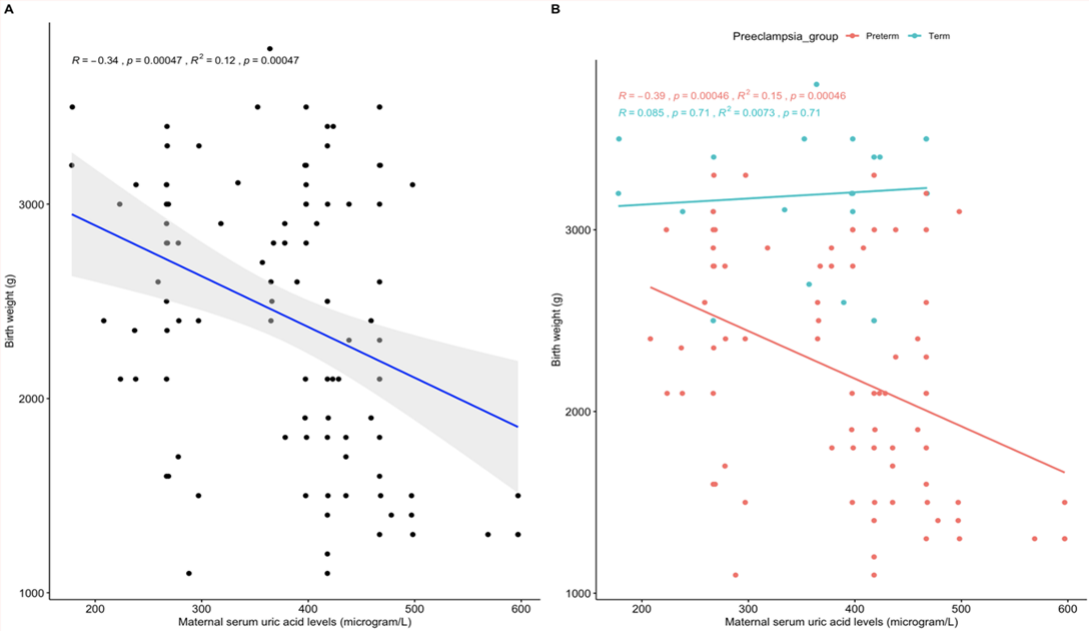
A,B) correlation between maternal serum uric acid levels and birth weight in pre-eclampsia

**Association between maternal serum uric acid level and birth weight:** Figure 1 shows the association between maternal serum uric acid level in pre-eclampsia and birth weight. In the overall pre-eclampsia study population (graph A in Figure 2), there was a significant negative association between maternal serum uric acid levels and birth weight (Pearson R= -0.34, p-value < 0.001). Further stratification (graph B in Figure 2), showed that the negative association was only significant in the preterm pre-eclampsia group (Pearson R= -0.39, p-value <0.001) compared with the term pre-eclampsia (Pearson R= 0.085, p-value <0.71)

**Figure 3:**
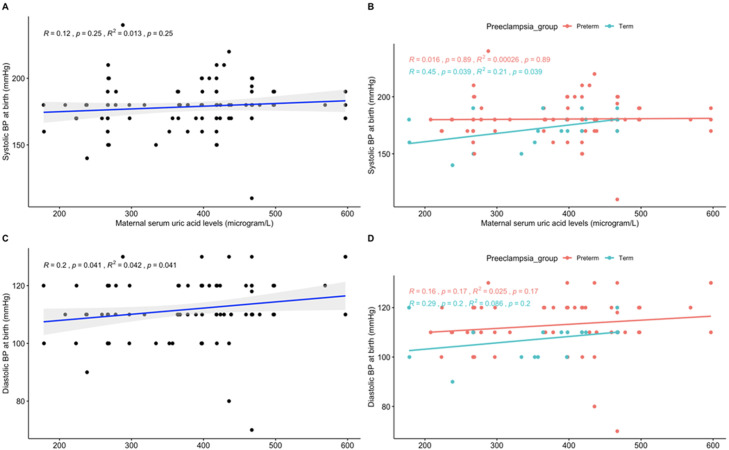
A,B,C,D) correlation between maternal serum uric acid levels in pre-eclampsia and systolic and diastolic blood pressure at birth

**Association between maternal serum uric acid level and blood pressure:** Figure 3 demonstrates the correlation between maternal serum uric acid levels in pre-eclampsia and blood pressure (diastolic and systolic). The overall pre-eclampsia study population had no significant association between maternal serum uric acid levels and systolic blood pressure at birth (Pearson R= 0.12, p-value =0.25) (graph A in Figure 3). Further stratification (graph B in Figure 3) showed a moderate positive association in the term pre-eclampsia (Pearson R= +0.45, p-value < 0.039) compared with the preterm pre-eclampsia (Pearson R= 0.016, p-value < 0.89). Also, there was a weak positive association between maternal serum uric acid levels and diastolic blood pressure at birth (Pearson R= 0.2, p-value = 0.041) in the total pre-eclampsia study population (graph C in Figure 3). Subgroup analysis (graph D in Figure 2) showed no statistical significance in both the term and preterm pre-eclampsia. Table 3 shows the results of the unadjusted and adjusted multivariable logistic regression model examining the association between maternal serum uric acid levels (hyperuricemia) and low birth weight, mode of childbirth, and blood pressure. Hyperuricemia was associated with low birth weight [aOR: 3.222 (95%CI: 1.098, 10.393)], caesarean section [aOR: 2.281 (95%CI: 1.084, 7.568)], and severe diastolic pressure at childbirth [aOR: 3.517 (95%CI: 1.123, 11.939)] adjusting for confounders.

**Table 3:** association between hyperuricemia in pre-eclampsia and low birth weight, mode of delivery, and blood pressure

Variable	OR	95%CI	P-value	aOR	95%CI	P-value
**Low birth weight^a^**	Ref 2.404	1.062, 5.605	0.038	Ref 3.222	1.098, 10.393	0.039
No						
Yes						
**Mode of delivery^a^**	Ref 3.099	1.257, 7.911	0.015	Ref 2.281	1.084, 7.568	0.035
Vaginal						
Caesarean						
**sBP at birth^b^**	Ref 2.210	0.461, 11.770	0.318	Ref 2.117	0.381, 13.166	0.388
Mild						
Severe						
**dBP at birth^c^**	Ref 3.429	1.237, 10.169	0.020	3.517	1.123, 11.939	0.034
Mild						
Severe						

sBP=systolic blood pressure; dBP=diastolic blood pressure; ^a^adjusted for maternal age, parity, gestational age at birth, diastolic and systolic blood pressure at diagnosis; ^b^adjusted for maternal age, parity, gestational age at birth, and systolic blood pressure at diagnosis; ^c^adjusted for maternal age, parity, gestational age at birth, and diastolic blood pressure at diagnosis

## Discussion

In this study, important clinical associations between maternal serum uric acid and perinatal outcomes in pre-eclampsia were evaluated. Hyperuricemia occurred in 61% of the pre-eclamptic population with no significant difference between preterm and term categories. We found a significant negative association between maternal serum uric acid levels and birth weight in pre-eclampsia with the significance limited to preterm only but not term pre-eclampsia. The mean gestational age at diagnosis was significantly lower in preeclamptic women with hyperuricemia compared with those with normal levels of uric acid. Hyperuricemia was significantly associated with low birth weight, caesarean section, and severe diastolic pressure during childbirth.

Hyperuricemia is considered predictive of severe pre-eclampsia, adverse pregnancy outcomes, and increased risk of cardiovascular and renal complications [7,19]. In this study, hyperuricemia was associated with an increased risk of low birth weight. In China, Ryu *et al*. (2019) also reported a significant association between elevated maternal uric acid levels and low birth weight in pre-eclampsia [20] as observed in our study and that of Enaruna *et al*. in Nigeria [19] and Ababio *et al*. in Ghana [21]. Also, there was a significant negative correlation between maternal uric acid levels and birth weight in pre-eclampsia but the associated significance was limited to the preterm only but not term pre-eclampsia.

The association between hyperuricemia and risk of pre-eclampsia is well documented [22,23] and uric acid has been considered as a major determinant of pre-eclampsia severity [6,9]. In our study, there was no significant association between maternal uric acid and the severity of hypertension at the time of diagnosis. However, with disease progression, hyperuricemia was significantly associated with severe diastolic hypertension but not systolic blood pressure. Further stratification showed a significant positive correlation with systolic blood pressure at birth in term but not preterm pre-eclampsia. Our findings are consistent with that of Ryu *et al*. who reported serum uric acid levels were positively associated with severe systolic blood pressure [20]. In their study, a uric acid cut-off of 377.7 microgram/l (6.35mg/dl) was used in predicting low birth weight similar to the 375 microgram/l used in our study. More recently, Nahar *et al*. reported a higher serum uric acid level in severe pre-eclampsia compared to the mild group and a positive association with the severity of hypertension [24]. Similar findings of significant elevation of serum uric acid levels in severe PE were reported by Asgharnia in Iran [25]. In a recent meta-analysis, Bellos *et al*. concluded that maternal serum uric acid levels are elevated in pre-eclampsia and can be predictive of disease severity and pregnancy complications [12].

In this study, caesarean delivery occurred in 81.7% of the pre-eclamptic women with hyperuricemia compared with 59% in women with normal uric acid levels. The risk of caesarean section associated with hyperuricemia in pre-eclampsia was doubled (OR= 2.281) compared to the normo-uricemia group. In a similar study, Gowri and Al-Zakwani reported a significantly higher probability of caesarean birth in association with pre-eclampsia complicated with hyperuricemia (33% versus 12%) [26]. However, in women with gestational hypertension or mild pre-eclampsia, van der Tuuk *et al*. found an increased risk of caesarean section with a lower odds ratio of 1.4 [27] using the data from the HAPITAT trial [28]. In a previous study at the same tertiary center [29], nearly 60% of women with pre-eclampsia delivered via caesarean section, lower than the 78% reported in the current study. However, in this study, hyperuricemia was found to be significantly associated with an increased caesarean section rate. This finding may be attributed to the hypothesized close association between hyperuricemia and pre-eclampsia disease severity which might have necessitated urgent delivery to prevent adverse pregnancy outcomes.

In summary, there is still a lack of consensus on the association between hyperuricemia in pre-eclampsia and pregnancy complications [8,9]. Whilst some studies found a significant association between hyperuricemia and adverse pregnancy outcomes [10] others did not. For instance, Priya *et al*. reported a positive association between hyperuricemia and fetal but not maternal complications [11]. The main strength of this study is the determination of the association of maternal uric acid levels in pre-eclampsia and pregnancy outcomes which might be clinically useful as an adjunctive tool predicting severe disease. The main limitation of the study relates to the cross-sectional design employed and the relatively small sample size. Despite the stated limitations of the study, the findings of this analysis provide useful information in the clinical management of pre-eclampsia in the country.

## Conclusion

Hyperuricemia in pre-eclampsia was significantly associated with low birth weight, caesarean section, and severe diastolic blood pressure at birth. There was a significant negative association between maternal serum uric acid levels and birth weight in preterm but not term pre-eclampsia. Hyperuricemia in pre-eclampsia may be clinically useful in predicting pregnancy outcomes, especially in preterm pre-eclampsia and the severity of hypertension. Further longitudinal study is recommended in exploring the clinical significance of maternal uric acid levels and the pregnancy outcomes in pre-eclampsia.

### What is known about this topic

- *Hyperuricemia is a common finding with severe pre-eclampsia and clinically precedes the development of hypertension and proteinuria;*
- *The association between serum uric acid and complications of pre-eclampsia is still conflicting*.

### What this study adds

- *Hyperuricemia in pre-eclampsia was significantly associated with low birth weight, caesarean section, and severe diastolic blood pressure at birth;*
- *There was a significant negative association between maternal serum uric acid levels and birth weight in preterm but not term pre-eclampsia*.
